# Reprogramming of the gut microbiota following feralization in *Sus scrofa*

**DOI:** 10.1186/s42523-023-00235-x

**Published:** 2023-02-24

**Authors:** Simona Petrelli, Maria Buglione, Eleonora Rivieccio, Ezio Ricca, Loredana Baccigalupi, Giovanni Scala, Domenico Fulgione

**Affiliations:** 1 Department of Biology, University of Naples Federico II, Via Cinthia 26, 80126 Naples, NA Italy; 2 Department of Humanities, University of Naples Federico II, Via Porta Di Massa 1, 80133 Naples, Italy; 3 Task Force On Microbiome Studies, University of Naples Federico II, 80100 Naples, NA Italy; 4 Department of Molecular Medicine and Medical Biotechnology, University of Naples Federico II, Via Pansini 5, 80131 Naples, NA Italy

**Keywords:** *Sus scrofa*, Gut microbiota, Reprogramming, Feralization

## Abstract

**Background:**

Wild boar has experienced several evolutionary trajectories from which domestic (under artificial selection) and the feral pig (under natural selection) originated. Strong adaptation deeply affects feral population’s morphology and physiology, including the microbiota community. The gut microbiota is generally recognized to play a crucial role in maintaining host health and metabolism. To date, it is unclear whether feral populations’ phylogeny, development stages or lifestyle have the greatest impact in shaping the gut microbiota, as well as how this can confer adaptability to new environments. Here, in order to deepen this point, we characterized the gut microbiota of feral population discriminating between juvenile and adult samples, and we compared it to the microbiota structure of wild boar and domestic pig as the references. Gut microbiota composition was estimated through the sequencing of the partial 16S rRNA gene by DNA metabarcoding and High Throughput Sequencing on DNA extracted from fecal samples.

**Results:**

The comparison of microbiota communities among the three forms showed significant differences. The feral form seems to carry some bacteria of both domestic pigs, derived from its ancestral condition, and wild boars, probably as a sign of a recent re-adaptation strategy to the natural environment. In addition, interestingly, feral pigs show some exclusive bacterial taxa, also suggesting an innovative nature of the evolutionary trajectories and an ecological segregation in feral populations, as already observed for other traits.

**Conclusions:**

The feral pig showed a significant change between juvenile and adult microbiota suggesting an influence of the wild environment in which these populations segregate. However, it is important to underline that we certainly cannot overlook that these variations in the structure of the microbiota also depended on the different development stages of the animal, which in fact influence the composition of the intestinal microbiota. Concluding, the feral pigs represent a new actor living in the same geographical space as the wild boars, in which its gut microbial structure suggests that it is mainly the result of environmental segregation, most different from its closest relative. This gives rise to interesting fields of exploration regarding the changed ecological complexity and the consequent evolutionary destiny of the animal communities involved in this phenomenon.

**Supplementary Information:**

The online version contains supplementary material available at 10.1186/s42523-023-00235-x.

## Background

The domestication of wild boar (*Sus scrofa*) is an evolutionary process started in the Neolithic age [1, 2], continued even in the following millennia [3]. During this period, swine have undergo a strong selection for human-desirable traits [4–6] resulting in a variety of different phenotypes affecting brain size, coat color, sexual maturity, growth rate, body size, and behavior [4, 7–14].

After domestication, some pig populations have experienced the so-called feralization process. Feral pigs are domesticated animals that, due to either accidentally or intentionally human-mediated events [15], return to the wild manifesting some pre-domestication traits [16–19], although feralization cannot be considered a simple reversal of domestication [20]. Feralization is a complex process and brings together populations that were at different times returned to the wild. The number of generations that domestic pigs spend in the wild affects the phenotype due to the different pressures of natural selection. One of the main variations associated with these conditions is the source of food, directly connected to the shape of the intestine [21] and its microbiota community [22]. Pig heavily depends on humans for food and water while wild forms obtain resources autonomously, supported by their plasticity and adaptability [14, 23, 24]. Feral pigs, with a domestic past, more or less distant, adapt to diverse and discontinuous food sources over time and the microbiota adaptation is expected.

Gut microbiota is involved in many vital processes for the host, including metabolism [25, 26], behavior [27], and immune system [28]. Hosts and their microbiota community co-evolve toward mutualism and homeostasis [29], and their relationships are strongly influenced by host-related factors such as age, sex, genotype, habitat, and lifestyle [30, 31].

Previous work on domestic animals shows that piglets born with an almost sterile intestinal tract, which is gradually populated by bacteria from the environment [32]. Gut microbiota development as a function of age is therefore an inevitable step. What is less clear is the definition of the principles of age-dependent microbiota development. This topic becomes even more intriguing and with evolutionary implications if we consider the feral pig that grow in the wilderness, like its ancestral form, the wild boar.

The regained wild diet and the interaction with a more complex environment is correlated with a reorganization of the gut microbiota in feral pigs, that show own unique bacterial community [19]. The dynamic underlining this remodeling could be influenced by factors related to the inheritance, development stages and/or lifestyle. Indeed, microbiota may affect host evolution by amplifying its trophic niche, thereby promoting adaptive trajectories, influencing the evolution of phenotypic plasticity, and affecting fitness via selection on traits relating to microbiota composition [33–37].

Here, in order to reveal the drivers reshaping the gut microbiota during feralization, we compare the gut communities of juvenile and adult feral pigs, between each other, and with those of domestic pig and wild boar.

## Materials and methods

### Study area and sampling

The study site was selected to have populations of wild boars, feral pigs and farms raised pigs in the closest areas. In particular, it includes the upland of Golgo and Supramonte Mountain in the Eastern part of the Sardinia Island (Italy, 40°5′21″N–9°40′2″E) (Additional file 1: Fig. S1).

Considering the variability in *S. scrofa*, we assigned our samples to three different forms on bases of phenotypic traits, behavior, and environmental segregation: (i) Sardinian wild boar (WB), showing a typical wild phenotype without any intentional contact with humans; (ii) domestic pig (DP), artificially selected breeds; (iii) the feral pig (FP), living free in remote areas, in sympatry with wild boar, and only sporadically in contact with their herders. We classified feral pig samples according to the age into juvenile (< 1 years) and adult (> 1 years).

The reference microbial communities of wild boars and pigs were sampled in individuals aged at least one year old (based on body size and coat color) to characterize the mature communities. This was assumed since we were interested in detecting the variation in the “feral experimental group” and considering the others two groups as controls in this experimental design.

The good health of animals was evaluated by a veterinary attending our sampling procedures. A total of 35 fecal samples were collected immediately after observation of animal defecation, without disturbing them (Wild boars, WB: N = 7; Domestic pigs, DP: N = 10; Juvenile feral pigs, FPJ: N = 8; Adult feral pigs, FPA: N = 10). Feral pigs were aged on the basis of herders information.

To avoid ground contamination from bacterial soil we collected the topper layer of the feces that has not touched the ground. Samples were handled with sterilized equipment, placed in sterile tubes with of 99.6% ethanol and then immediately stored at − 20 °C, transported at controlled temperature to the laboratory and processed for the DNA extraction and sequencing, at most three days after sampling.

### DNA extraction from fecal samples

The DNA extraction from fecal materials was performed with QIAamp DNA Fast Stool Mini Kit (QIAGEN GmbH Valencia, CA, USA), according to guidelines’ recommendation. To check for potential contaminations, blank extractions were systematically included. Finally, the quality and quantity of extracted DNAs were evaluated using Nanodrop ND-2000 (Nanodrop, Wilmington, DE, USA) and Qubit Fluorometer 3.0 (Thermo Fisher Scientific).

### Miseq sequencing of a partial 16S rRNA gene

A fragment of about 190 bp of 16S rRNA gene sequence was amplified using Probio_Uni (5′-CCTACGGGRSGCAGCAG-3′) and /Probio_Rev (5′-ATTACCGCGGCTGCT-3′) primers, targeting the V3 region, and sequenced on Illumina MiSeq platform by GenProbio srl (www.genprobio.com) according to [38]. The sequencing also included blank-negative water samples and specific mock communities (ZymoBIOMICS HMW DNA Standard) as additional quality check control.

After demultiplexing, the reads were trimmed and filtered to remove low quality raw data and chimeras. All quality-approved reads were exported as.fastq files and processed using a script based on the QIIME software suite [39]. Paired-end reads pairs were assembled to reconstruct the complete Probio_Uni / Probio_Rev amplicons. Quality control retained those sequences with a length between 140 and 400 bp and mean sequence quality score > 20. Sequences with homopolymers > 7 bp and mismatched primers were omitted.

The reads obtained from the sequencing were also filtered for Eukaryotic, Mitochondrial and Chloroplast sequences.

In order to calculate alpha and beta diversity indices, 16S rRNA Operational Taxonomic Units (OTUs) were defined at ≥ 99 sequence homology using DADA2 [40] and OTUs not encompassing at least 2 sequences of the same sample were removed. All reads were classified to the lowest possible taxonomic rank using QIIME2 [39] and the SILVA database v. 132 as reference dataset [42].

Finally, biodiversity of the samples was calculated considering the number of observed OTUs, Chao1 and Shannon indexes to estimate the sequencing depth.

Beta diversity was calculated as a Principal Coordinates Analysis (PCoA) using Bray–Curtis dissimilarity [43] on bacterial genera in PAST v 3.2. software [44]. Data have been processed to obtain a multivariate heatmap in R using packages "fields" [45] and "MBA" packages.

The alpha diversity descriptors (within sample diversity), Richness (S), and Shannon (H) indices, were calculated in Past3 software, considering the bacterial genera among the three forms.

### Statistical analyses

The symmetrical Venn diagram (free Venn Diagram Tool [46]) was performed for the identification of exclusive and shared bacterial genera among the microbiota.

In our dataset, core microbiomes were quantified based on the occurrence of genera within the samples collected, setting a cut-off of 30% [47, 48]. To test for significant differences of alpha diversity among the groups, we performed a Kruskal–Wallis test, followed by pairwise Wilcoxon tests in R [49].

One-way PERMANOVA test was performed to evaluate the significance of multivariate analyses in Past 3 software [44] and a *p*-value of less than 0.05 was considered statistically significant.

#### Bacterial associates according to categories

To pinpoint which taxa may play a role in feral pig developmental stage (juvenile *vs* adult) and in its references (wild boar and domestic pig), we identified bacterial associates that represent the bacterial OTUs whose ecology or function is likely important for the host. We determined the bacterial associates via effect size analysis (LEfSe, [50]) using the default settings.

#### Functional profile of gut bacterial community

We explored if the functional profile of the gut bacterial community showed differences between categories. The metabolic properties of the microbial communities were predicted using PICRUSt2 (version 2.3.0b) [51]. We tested with PERMANOVA if metabolic function abundances varied between juvenile and adult feral pig, as well as, *versus* both wild boars and domestic pigs.

## Results

### Sequencing reports

From 35 fecal samples (wild boars, WB: N = 7; domestic pigs, DP: N = 10; juvenile feral pigs, FPJ: N = 8; and adult feral pigs, FPA: N = 10), a total of 2,253,218 reads were generated from sequencing (mean value: 64,378 ± 10,084) and after the quality filtering, a total of 1,988,359 reads were obtained (mean value: 56,810 ± 9,087). The sample with the lowest representation gave 35,225 reads. The total number of identified OTUs was 6,254 and OTUs not encompassing at least 2 sequences of the same sample were removed.

The analysis of blank-negative water samples and mock communities’ controls did not reveal any significant differences compared to the expected profiles, and blank-negative water samples did not show any bacterial profile.

The Observed OTUs, Chao1, and Shannon rarefaction curves reached the plateau for all samples, showing that the sequencing depth was sufficient for capturing a majority of microbial diversity and differences in microbial communities in the samples (Additional file 2: Fig. S2 a–c).

### Microbiota comparison at phylum, family and genus level

Considering the taxa with a relative abundance > 1%, we identified 15 phyla (Additional file 3: Table S1), 60 families (Additional file 4: Table S2) and 138 genera (Additional file 5: Table S3).

The development from a juvenile to adult microbial community in the feral pig appears to leave the phyla unchanged with the exception of Fibrobacteres, more evident in the adult. Finally, *Spirochaetes* is the third most abundant, both in juvenile and adult feral pigs (4.238% ± 1.907 in FPJ; 5.407% ± 3.024 in FPA).

**Fig. 1:**
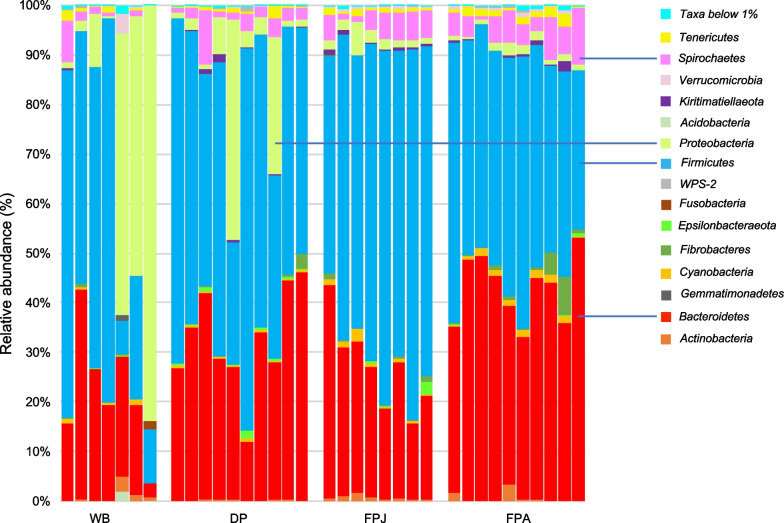
Relative abundance (%) of phyla in each sample. The bars in the graph show the proportion of the sequences in each sample that can be classified at the phylum level. The blue lines indicate phyla that occur in at least one sample with a frequency of occurrence ≥ 10%. WB, wild boar; DP, domestic pig; FPJ, juvenile feral pig; FPA, adult feral pig

Extending the comparison to wild boar and pig, *Firmicutes* is on average the most abundant phylum (43.128% ± 28.863 in WB; 52.436% ± 15.609 in DP; 62.445% ± 9.576 in FPJ and 44.76% ± 7.334 in FPA) (Fig. 1, Additional file 3: Table S1), followed by *Bacteroidetes* (except for the wild boar, whose the second most abundant phylum is *Proteobacteria* 29.655% ± 34.072), which almost doubles in the transition from juvenile to adult feral pigs (26.452% ± 8.653 in FPJ and 42.34% ± 7.259 in FPA).

**Fig. 2:**
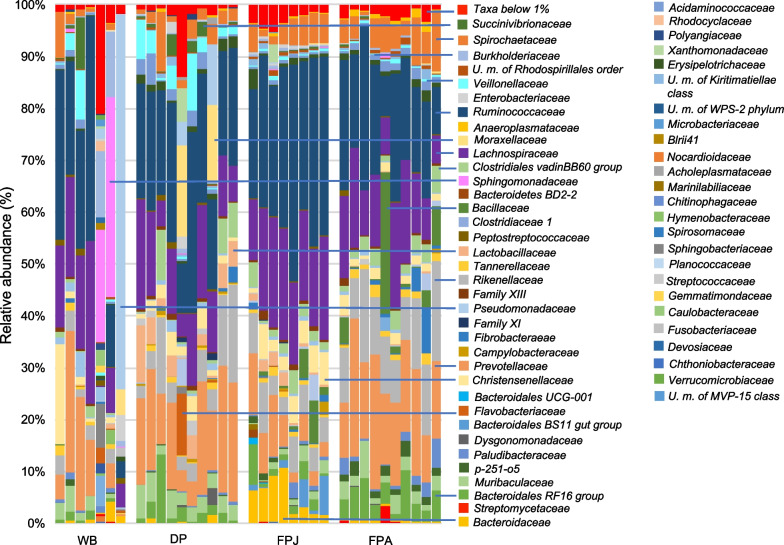
Relative abundance (%) of family in each sample. The bars in the graph show the proportion of the sequences in each sample that can be classified at the family level. The blue lines indicate families that occur in at least one sample with a frequency of occurrence ≥ 10%. WB, wild boar; DP, domestic pig; FPJ, juvenile feral pig; FPA, adult feral pig

Screening at family level, bacterial communities depict a new pattern of diversity (Fig. 2, Additional file 4: Table S2). In the microbiota of adult feral pigs we found a switch between dominant and subdominant groups compared with juvenile feral pigs. Indeed significantly decrease *Ruminococcaceae* (19.3% ± 7.273) and *Lachnospiraceae* (13% ± 5.518, but not significantly) while *Prevotellaceae* (17.1 ± 5.859) shows a significant increase (ANOVA followed by pairwise Tukey's Honest Significant Difference tests, Table 1 and Fig. 2).

**Table 1:** Analysis of Variance (ANOVA) and Tukey test between juvenile (FPJ) and adult feral pigs (FPA)

	Sum of Squares	df	Mean Square	F	*p*
***Ruminococcaceae***					
Between groups	0.0621	1	0.0621	12.5395	0.002717
Within groups	0.0793	16	0.005		
Total	0.1414	17			
***Lachnospiraceae***					
Between groups	0.0106	1	0.0106	4.73555	0.051852
Within groups	0.0357	16	0.0022		
Total	0.0463	17			
***Prevotellaceae***					
Between groups	0.0511	1	0.0511	19.53221	0.000429
Within groups	0.0419	16	0.0026		
Total	0.093	17			

The juvenile feral pigs are similar to domestic pigs, in particular for *Ruminococcaceae* (31.125% ± 6.728), *Lachnospiraceae* (17.875% ± 3.441), *Prevotellaceae* (6.375% ± 3.962).

Considering the taxonomic resolution at genus level (Additional file 5: Table S3), we found a decrease in the number of bacterial genera in the transition from juvenile to adulthood in feral pigs (120 genera in FPJ, and 110 in FPA).

In the juvenile feral pig, the most abundant genera are represented by the *Faecalibacterium* (5.302% ± 3.558), *Ruminococcaceae UCG-005* (5.24% ± 3.798), and *Bacteroides* (5.038% ± 3.477). We found the only one similar dominant genus in domestic pig showing three most abundant genera: *Rikenellaceae RC9 gut group* (6.555% ± 6.118), followed by *Prevotella 9* (4.778% ± 3.392) and *Ruminococcaceae UCG-005* (4.355% ± 2.313).

This pattern of dominant genera can also be found in the feral adult with gut microbiota constituted by *Rikenellaceae RC9 gut group* (11.339% ± 3.956), followed by *Prevotellaceae NK3B31 group* (5.246% ± 2.092) and *Ruminococcaceae UCG-005* (5.145% ± 2.253).

**Fig. 3:**
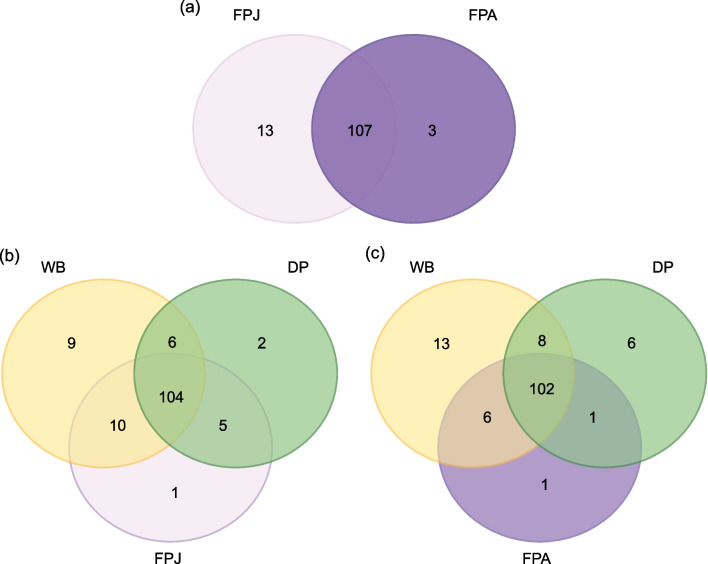
Symmetric Venn diagrams of genera (based on the presence / absence) of the gut microbiota. Comparison between juvenile (FPJ) and adult (FPA) feral pigs (a). Comparison among wild boar (WB), domestic pig (DP) and juvenile feral pig (FPJ) (b); comparison among wild boar, domestic pig and and adult feral pig (FPA) (c)

In the feral form, the definition of an adult microbiota is determined by the loss of exclusive genera, in fact for these suidae only 3 exclusive genera can be counted in adulthood towards 13 of the youthful age (Fig. 3a, Additional file 6: Table S4).

The core microbe of the four categories (WB, DP, FPJ and FPJ) was obtained considering those bacterial genera that occurred in at least 30% of samples. From this comparison emerged that the microbial core of suidae is composed of 103 bacterial genera (Additional file 7: Table S5).

These communities characterized by different age compared with wild boars and domestic pigs showed that the juvenile feral pigs share 5 genera with the domestic pigs, while, in the same context, these go down to 1 genus considering the adult feral pigs (Fig. 3b-c). A similar pattern is observed in the comparison with wild boars.

The number of genera shared among all these categories and the number of exclusive genera in the feral pigs, remains rather similar if juvenile and adult are used in the comparisons (Fig. 3b-c, Additional file 8: Table S6, Additional file 9: Table S7).

### Diversity within and between categories

**Fig. 4:**
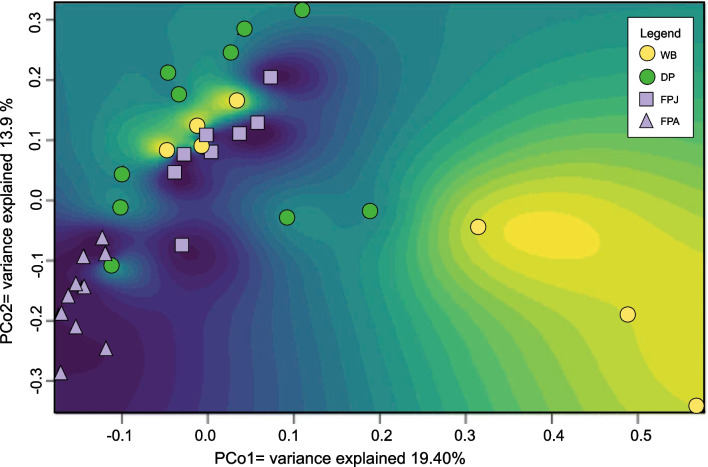
Principal Coordinate Analysis (heatmap PCoA) plot generated based on Bray–Curtis. Each point represents the intestinal microbiota of an individual. A buffer was generated around each of the microbial communities to characterize the multivariate space. Wild boar, WB; domestic pig, DP; juvenile feral pig, FPJ; adult feral pig, FPA

Multivariate Analysis shows mutually well-characterized bacterial communities using the information at genus level. To interpret the relative position of the objects, we developed a heatmap with intensities determined by the position of each microbiotic communities examined. The area that characterized the microbiota community of feral pigs (FP) is dark blue, in which it is possible to distinguish the two diverse groups (juvenile and adults). The juvenile microbiota (FPJ) clusters in the upper left with some points of overlap with the microbiota of wild boar and domestic pig (around the origin of the graph), while the microbiota of adult forms (FPA) localizes in the negative value of the two axes (Fig. 4). Interestingly, the feral pigs are distributed in the delimited and equidistant area between the two references (wild boar and domestic pigs). It is possible to identify a yellow area, which characterizes the wild boar communities (WB), extending from the center to the lower right portion of the graph (Fig. 4). The green area identifies the microbiota from domestic pig, concentrated in the upper left portion of the multivariate space, showing an overlapping area with the wild boar.

We also evaluated the significance of this spatial path by one-way PERMANOVA test which returned values that were always significant (F: 4.165; *p*: 0.0001) (Additional file 10: Table S8).

**Fig. 5:**
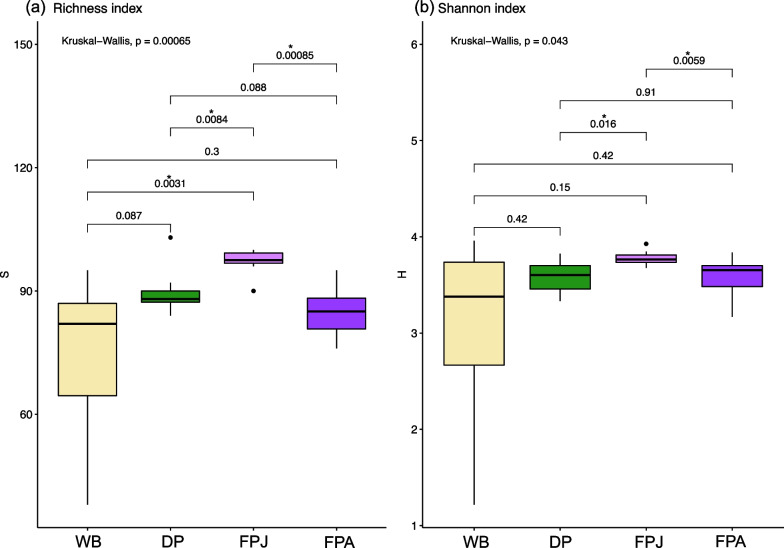
Alpha-diversity. (a) Richness (S) and (b) Shannon (H) indices, for wild boar (WB), domestic pig (DP), and juvenile (FPJ) and adult (FPA) feral pigs. Asterisks indicate significant differences between categories

From the analysis of alpha-diversity descriptors statistically significant differences were observed for both Richness (S) (Kruskal–Wallis, p: 0.00065) and Shannon (H) indices (Kruskal–Wallis, p: 0.043) (Fig. 5a, b). In particular, juvenile feral pigs seem to introduce greater diversity than adult feral pigs (Fig. 4a, b). The adult feral pig shows a similar diversity compared with wild boar and domestic pig meanwhile for the juvenile it increases (Fig. 5a, b).

**Fig. 6:**
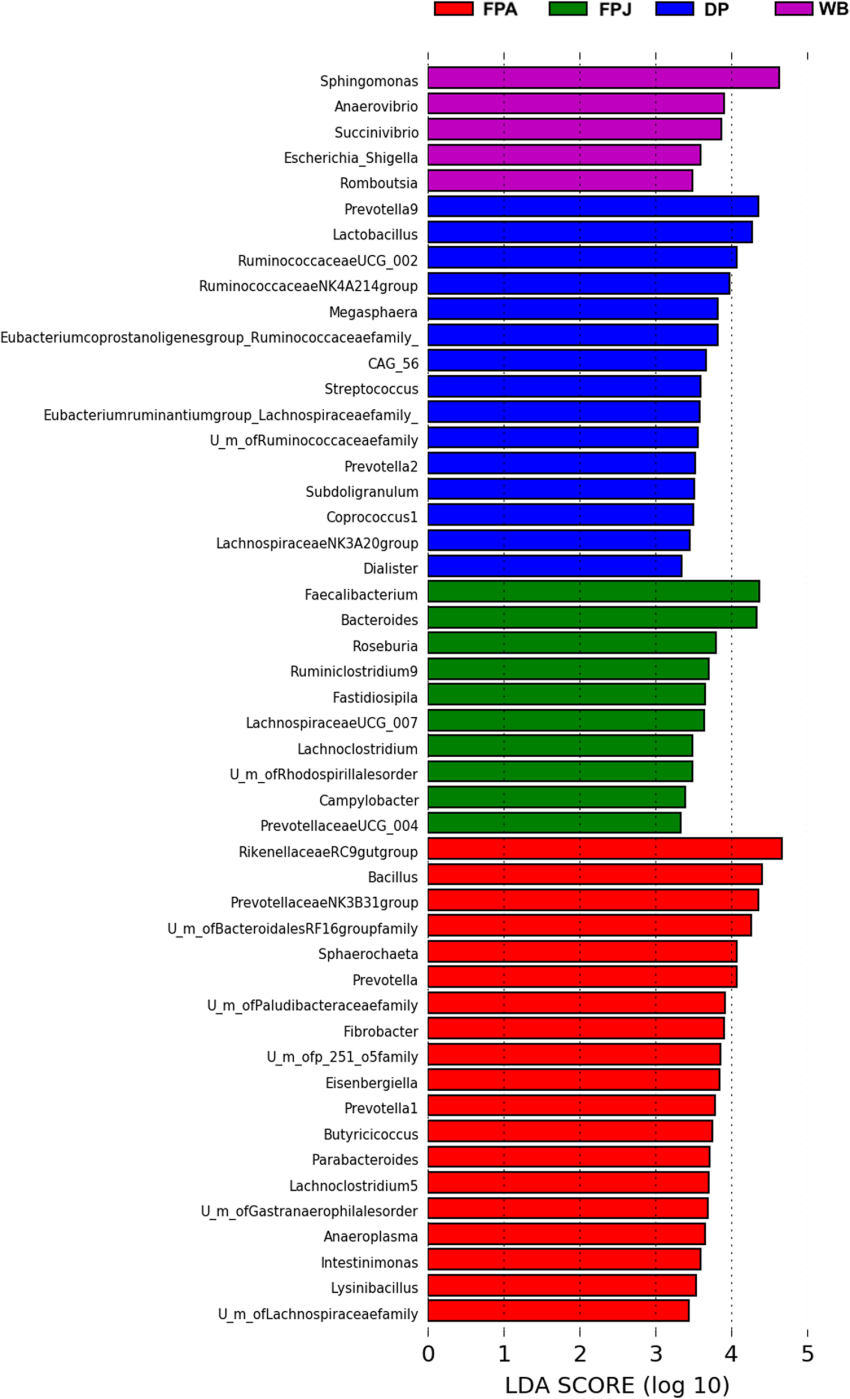
LEfSe analysis. Only pathway with an LDA significance threshold > 2 are shown. Wild boar (WB), domestic pig (DP),  juvenile (FPJ) and adult (FPA) feral pigs

Linear discriminant Analysis Effect Size analysis (LEfSe) detected 49 bacterial associates with the different categories, of which 10 and 19 were more abundant in FPJ and FPA, respectively. Wild boar showed 5 associated bacterial and 15 were found in domestic pig (Fig. 6; Additional file 11: Table S9).

The PICRUSt2 analysis from the metabolic function of the bacteria exclusive to each group, as evidenced by Lefse analysis, showed a similarity between juvenile feral pigs and domestic pigs, example given by L-lisyne biosynthesis VI and aerobic respiration 1 (cytochrome C). The wild boar was the most distant group considering of the metabolic functions of the bacteria exclusive to each group. Adult feral seem to cluster in an intermediate position, closer to wild boar than the other two (Additional file 12: Fig. S3).

## Discussion

In the complex host-associated microbial communities, the ability, and the speed of response to natural selection, depend on both adaptive plasticity and constraints deriving from the phylogenetic signal [52–54]. In light of this, the feral pig is an excellent model to explore this topic because it acts as an animal shaped by artificial selection (domestication) which must respond to the demands of natural selection.

Our observations and evolutionary deductions depend on the bacterial community structure. It can be analyzed in comparative terms through both change the bacterial abundance hierarchies and introduce new taxonomic entities. Indeed, some bacteria are simply modulated differently in feral forms and in the different two age categories, as shows in the comparison among two standard of both artificial selection (domestic pig) and natural selection (wild boar).

Proteobacteria, like other some taxa, is present in feral (juvenile and adult) and domestic pigs in negligible percentages, although segregates in the wild boar. This finding is rather unexpected if we consider the useful function of this bacterial group in the wildlife. Indeed, Proteobacteria plays a key role in preparing the gut for the colonization by the strict anaerobes required for healthy gut function [55]. Furthermore, it facilitates the degradation of lignin, and the high abundance of this phylum may be necessary to cope with the complex diet in wild [56].

Considering the bacterial communities at family taxonomic level, *Sphingomonadaceae* is totally absent in juvenile feral pigs and not very abundant in adult feral pigs and domestic pigs. *Sphingomonadaceae* are commonly isolated from soils, freshwater and marine habitats, activated sludge, plant phyllosphere or rhizosphere. Some are antagonistic against plant pathogens and induce plant growth promotion [57]. These two last observations suggest that the *Sphingomonadaceae* and *Proteobacteria* can be considered indicators of a wildlife style, with a diet deriving directly from the surrounding environment, plants, soil, and water. Its low abundance in the feral category is unexpected, but it may suggest a different ecological segregation from relative wild boar.

A different pathway was observed for *Bacteroidaceae*, which shows a decrease from wild boar to domestic pig and an increase in the feral pig. *Bacteroidaceae* family is an important indicator on the influence of the environment *versus* maternal contribution. It is known [32] that in domestic pigs there are two separate periods of life: before and after weaning. Milk nursing and the diet after weaning differ markedly and therefore affect the composition of pig gut microbiota. After weaning, *Bacteroidaceae* are replaced by *Prevotellaceae* (closely related families belonging to the order *Bacteroidales*). The switch from *Bacteroidaceae* to *Prevotellaceae* is quite rapid and takes < 1 week after weaning [58, 59]. The *Bacteroidaceae*/*Prevotellaceae* switch means that *Bacteroidaceae* are underrepresented in the microbiota of adult domestic pigs including sows, and these may act as an inappropriate source of *Bacteroidaceae* for their piglets [60]. Since the microbiota of piglets before weaning differs from the microbiota of adult pigs, the diet influences microbiota composition more than the contact between sow and piglets. In accordance with these observations carried out on domestic pigs, even our juvenile feral pigs show *Bacteridaceae* at 5.25% which then drops to less than 1% in adulthood (0.5%). As expected, the replacement with *Prevotellaceae* takes place with an increase from 6.4% in juvenile feral pigs to 17.1% in the adult ones, values comparable with those for wild boars and domestic pig categories. This *Bacteroidaceae*/*Prevotellaceae* switch suggests the strong effect of the environment in the definition of an adult microbial community in the feral pig and also an ancestral link to microbiota dynamic described for domestic pigs.

In general, the feral pigs, both juvenile and adult, share a higher number of genera with wild boars, suggesting that the feral population has long been in a wildness state. Furthermore, the juvenile feral pigs share a higher number of genera with domestic pigs than the adult ones (5 *versus* 1). However, the adult feral pig, does not converge towards a condition of microbiota similar to the wild boar, in fact, feral ranges from ten genera (juvenile) to 6 genera (adult) shared with wild boar.

Interpreting these results in an evolutionary key, we can hypothesize that feral populations, although born with a microbial community partially similar to that of the domestic pig, differ from the wild boar by acquiring a new and diverse condition.

The microbiota of adult feral pigs, therefore, is probably the adaptation to the wildlife, a more defined and less redundant community, on the basis of the bacterial groups most useful for exploiting natural resources. This also emerges comparing the juvenile from the adult microbiota. These two categories share 107 bacterial genera, but the number of exclusive genera drastically decreases from the juvenile to the adult condition. Accordingly, the adult microbiota is also the one with less richness.

The analysis of bacterial communities, assuming genera as variables and individuals as objects, orders wild boars and domestic pigs in a multivariate space where they are well discriminated, with wild boars close to high values of PCo2, and domestic pigs tending to stay close to high values of PCo1. Wild boars show extraordinary variation which may depend on the oldest individuals as reported by our field notes.

It is interesting to note how feral pigs are in an intermediate position with the two distinct age groups, and with juvenile showing affinity with the domestic and wild form. In particular, the similarities between juvenile feral and domestic pigs narrowed if we consider the metabolic function of the bacteria characterizing the experimental groups. The juveniles differ from the feral adults suggesting an interesting starting point for studying the adaptive plasticity of these animals.

## Conclusions

The microbiota of juvenile feral pigs is closer to the microbiota of the domestic form while the microbiota of the adults differs from both the domestic and the wild form. It seems to be adapted to the wild environment (in terms of structure and composition) as the wild boar, although in different way. However, it is important to underline that we certainly cannot overlook that these variations in the structure of the microbiota also depended on the different development stages of the animal, which in fact influence the composition of the intestinal microbiota.

This study sheds light on the relative contribution of heredity and environment to the evolution of the microbiota in suidae, although it highlights many questions that still need to be answered. Furthermore, according to our previous studies [5, 17, 19], our findings suggest that feral populations do not represent a simple reverse-domestication, but an independent line of evolution that readjusts some acquisitions deriving from the domestic (artificial selection) in a new wild context (natural selection).

The adaptability of feral swine, in the ability of colonizing wild environments (in a few generations), probably depends also on an ad hoc intestinal microbiota.

## Supplementary Information

**Additional file 1: Figure S1**. Study area. The red box indicates the investigated area

**Additional file 2: Figure S2**. Rarefaction curves for each sample: (a) Number of OTUs, (b) Chao 1 and (c) Shannon index

**Additional file 3: Table S1**. Phylum relative abundance

**Additional file 4: Table S2**. Family relative abundance

**Additional file 5: Table S3**. Genus relative abundance

**Additional file 6: Table S4**. Exclusive and shared bacterial genera between juvenile (FPJ) and adult feral pigs (FPA)

**Additional file 7: Table S5**. Core microbes of suidae

**Additional file 8: Table S6**. Shared and exclusive genera among wild boar (WB), domestic (DP) and juvenile feral pigs (FPJ)

**Additional file 9: Table S7**. Shared and exclusive genera among wild boar (WB), domestic (DP) and adult feral pigs (FPA)

**Additional file 10: Table S8**. One-way PERMANOVA test values

**Additional file 11: Table S9**. LEfSe analysis identification of bacteria genera in the four categories. Wild boar (WB), domestic (DP), juvenile feral pig (FPJ) and adult feral pig (FPA)

**Additional file 12: Figure S3**. The heatmap of metabolic pathways predicted by PICRUSt analysis in the four categories. Wild boar (WB), domestic (DP), juvenile feral pig (FPJ) and adult feral pig (FPA)

## Data Availability

The partial 16S rRNA gene sequences produced for this study are available in the Sequence Read Archive (SRA) under accession number PRJNA669575.

## Abbreviations

WB: Wild boar
DP: Domestic pigs
FPJ: Juvenile feral pigs
FPA: Adult feral pigs
