# Supplementary figures and images for: Reprogramming of the gut microbiota following feralization in Sus scrofa
Source: Anim Microbiome. 2023 Feb 24;5:14. doi: 10.1186/s42523-023-00235-x (PMC9951470; doi:10.1186/s42523-023-00235-x)

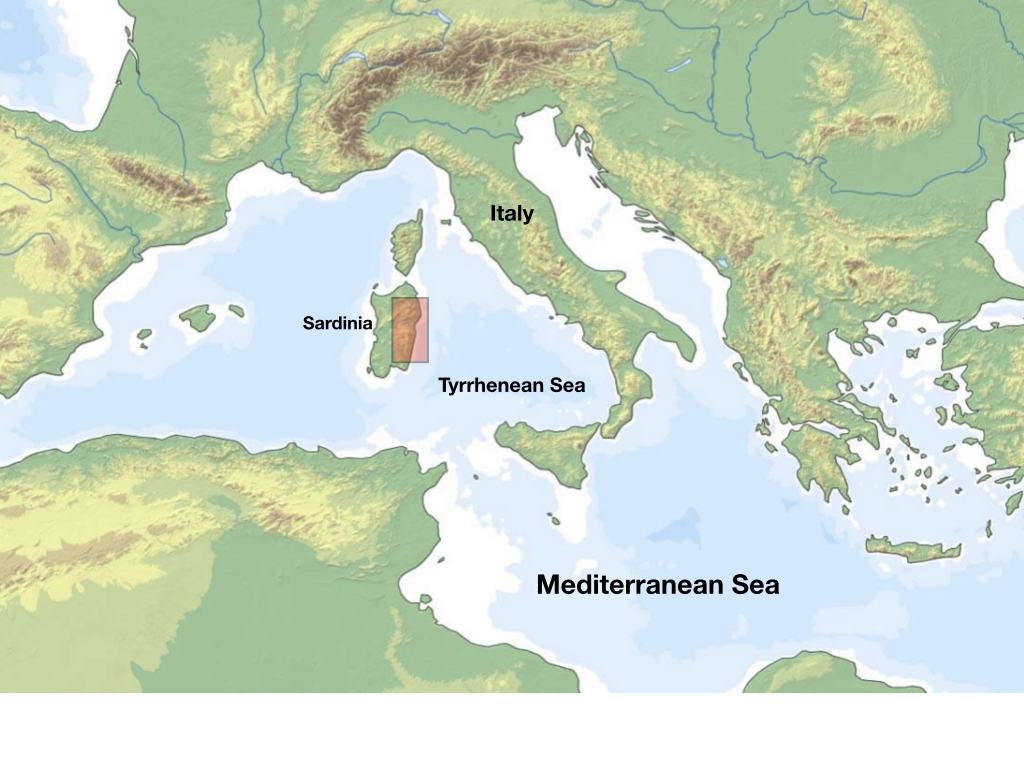

Supplement: Supplementary file 1 — Additional file 1: Figure S1. Study area. The red box indicates the investigated area [file 42523_2023_235_MOESM1_ESM.tiff]

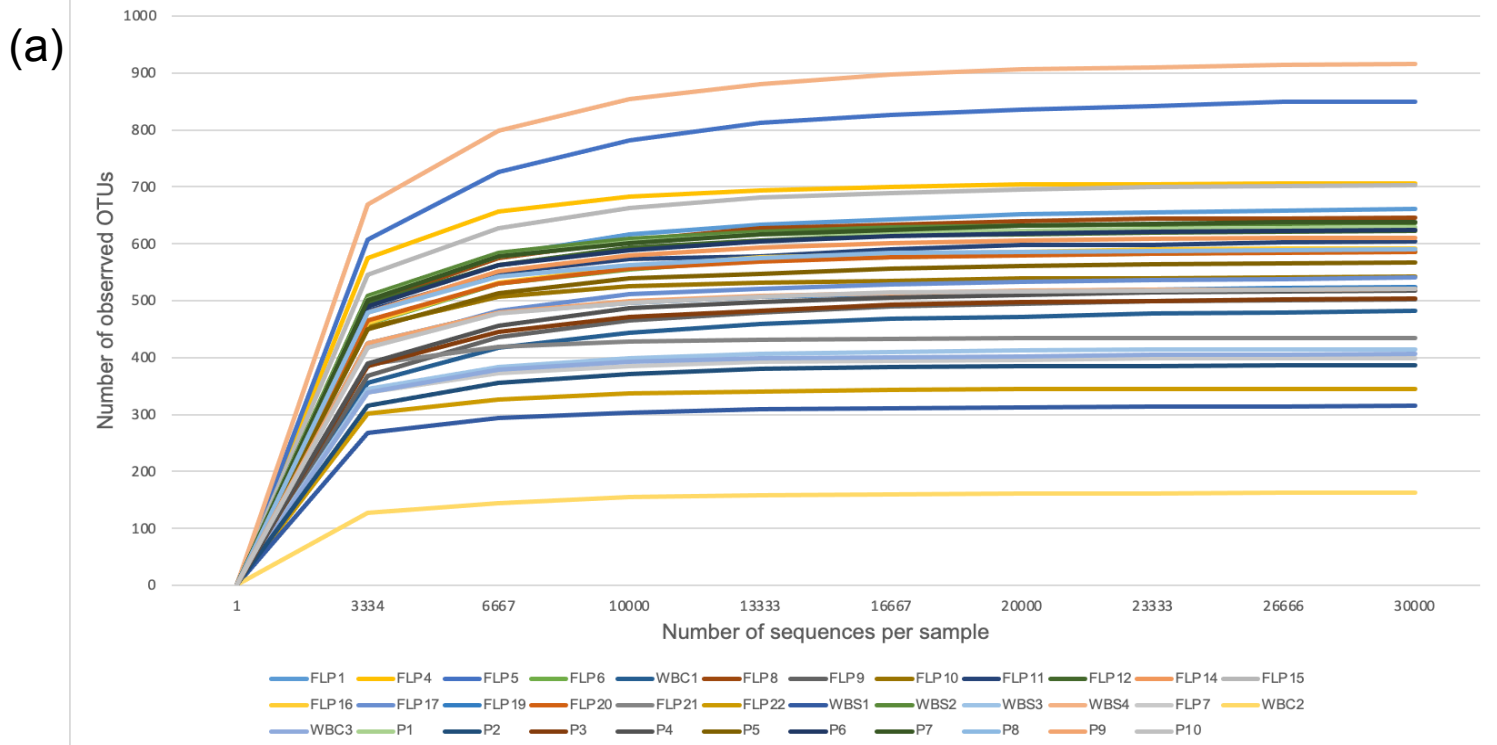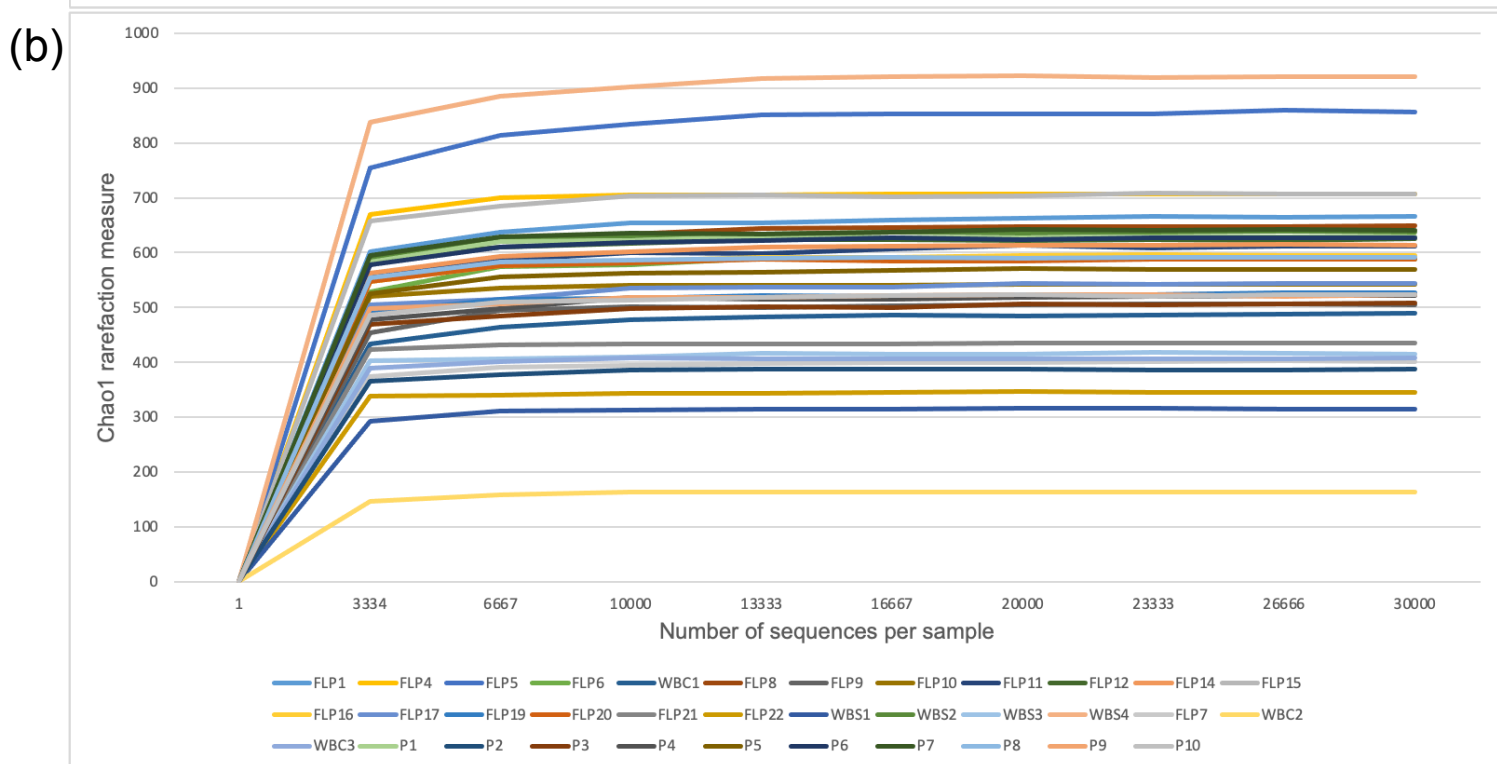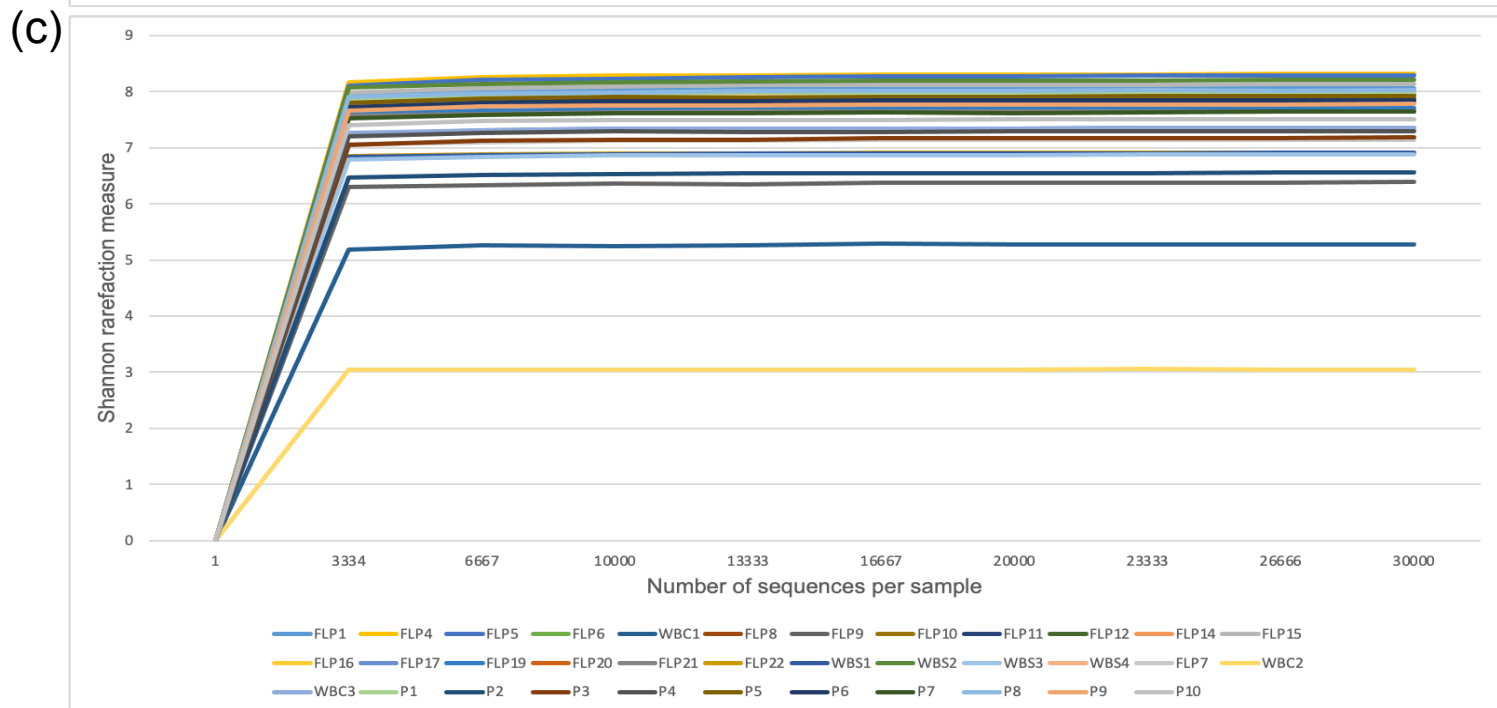

Supplement: Supplementary file 2 — Additional file 2: Figure S2. Rarefaction curves for each sample: (a) Number of OTUs, (b) Chao 1 and (c) Shannon index [file 42523_2023_235_MOESM2_ESM.pdf]

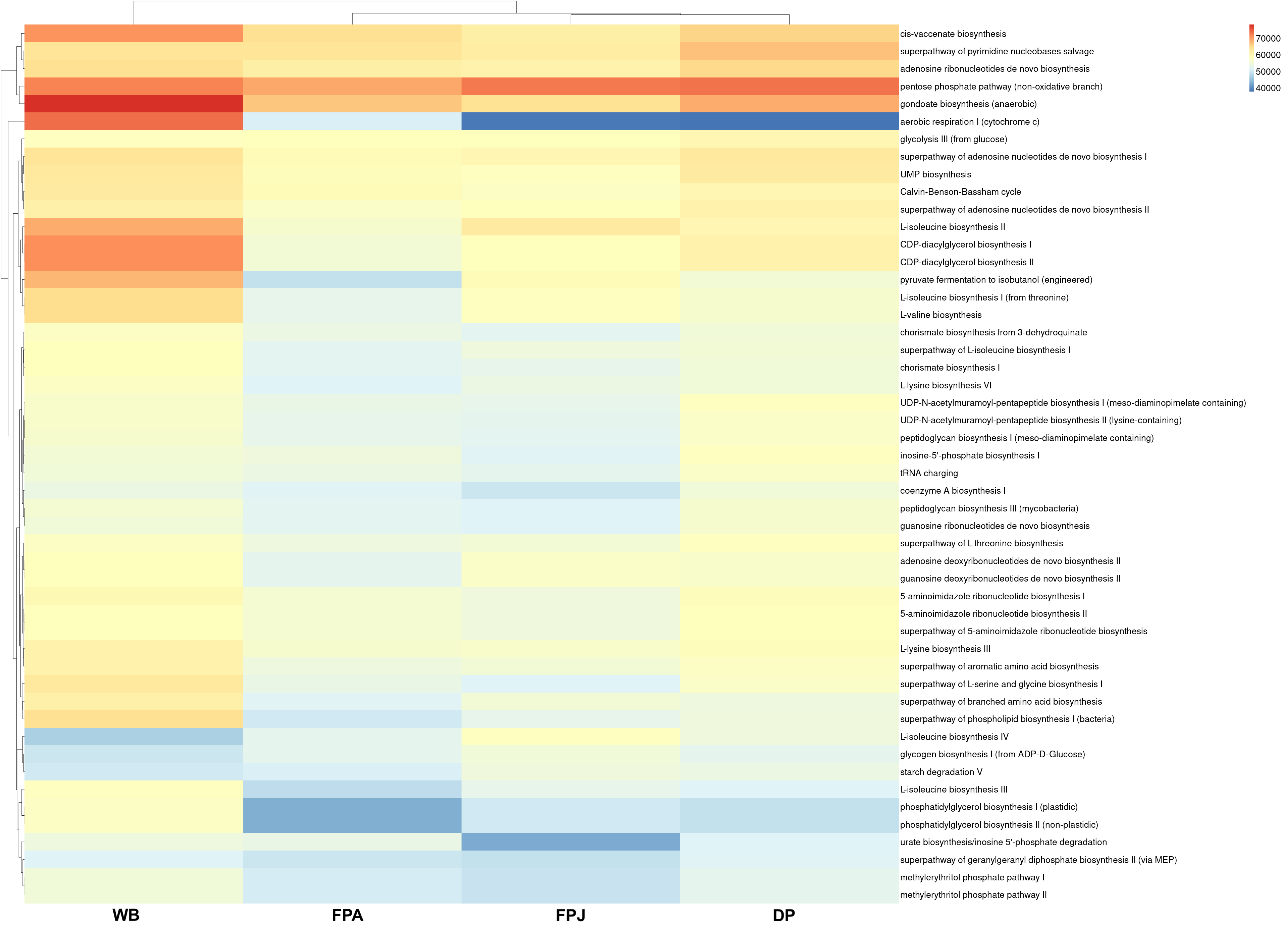

Supplement: Supplementary file 12 — Additional file 12: Figure S3. The heatmap of metabolic pathways predicted by PICRUSt analysis in the four categories. Wild boar (WB), domestic (DP), juvenile feral pig (FPJ) and adult feral pig (FPA) [file 42523_2023_235_MOESM12_ESM.png]
